# Unexpected monolayer-to-bilayer transition of arylazopyrazole surfactants facilitates superior photo-control of fluid interfaces and colloids†
†Electronic supplementary information (ESI) available: Synthesis and further characterization, videos of Marangoni flow. See DOI: 10.1039/c9sc05490a


**DOI:** 10.1039/c9sc05490a

**Published:** 2020-01-08

**Authors:** Christian Honnigfort, Richard A. Campbell, Jörn Droste, Philipp Gutfreund, Michael Ryan Hansen, Bart Jan Ravoo, Björn Braunschweig

**Affiliations:** a Institute of Physical Chemistry , Westfälische Wilhelms-Universität Münster , Corrensstraße 28/30 , 48149 Münster , Germany . Email: braunschweig@uni-muenster.de; b Center for Soft Nanoscience (SoN) , Westfälische Wilhelms-Universität Münster , Busso-Peus-Straße 10 , 48149 Münster , Germany; c Division of Pharmacy & Optometry , School of Health Sciences , University of Manchester , Oxford Road , Manchester M13 9PT , UK; d Institut Laue-Langevin (ILL) , 71 Avenue des Martyrs, CS 20156 , 38042 Grenoble Cedex 9 , France; e Organic Chemistry Institute , Westfälische Wilhelms-Universität Münster , Corrensstraße 40 , 48149 Münster , Germany

## Abstract

Interfaces that can change their chemistry on demand have huge potential for applications and are prerequisites for responsive or adaptive materials. We report on the performance of a newly designed *n*-butyl-arylazopyrazole butyl sulfonate (butyl-AAP-C_4_S) surfactant that can change its structure at the air–water interface by *E*/*Z* photo-isomerization in an unprecedented way. Large and reversible changes in surface tension (Δ*γ* = 27 mN m^–1^) and surface excess (Δ*Γ* > 2.9 μmol m^–2^) demonstrate superior performance of the butyl-AAP-C_4_S amphiphile to that of existing ionic surfactants. Neutron reflectometry and vibrational sum-frequency generation spectroscopy reveal that these large changes are caused by an unexpected monolayer-to-bilayer transition. This exceptional behavior is further shown to have dramatic consequences at larger length scales as highlighted by applications like the light-triggered collapse of aqueous foam which is tuned from high (>1 h) to low (<10 min) stabilities and light-actuated particle motion *via* Marangoni flows.

## Abstract 2

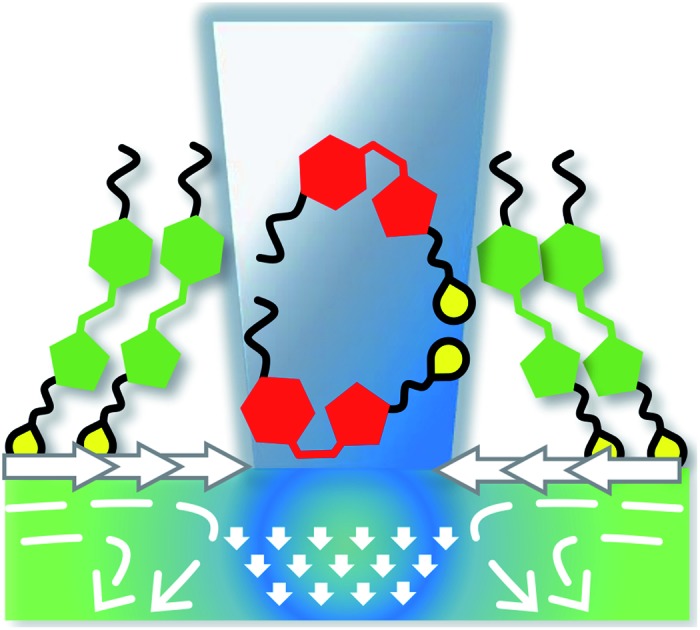
 *E*/*Z* photo-isomerization of a new surfactant causes substantial changes in interfacial properties, which are a prerequisite for responsive and adaptive material control on a molecular level.

## Introduction

Smart surfaces and interfaces can respond to external stimuli such as light, temperature or magnetic fields and change their physicochemical properties on demand. They have great potential to serve as hierarchical elements for responsive functional materials,^1^–^3^ light-induced actuation^4^,^5^ and have prospects to be developed further for adaptive materials with self-healing or self-learning functions. Prerequisites for such functions are distinct, precise and reproducible control of interfacial properties. Such control can be achieved by tuning molecular self-assembly through the use of an external stimulus.^2^,^6^ Here, soft interfaces are of great interest because they allow fast and reversible reconfigurations, *e.g.*, by de- or adsorption of molecules triggered by an external stimulus.^7^–^11^ For example, tuning the properties of fluid interfaces helps to develop smart foams with self-healing and adaptive functions.^3^,^12^ In addition, Marangoni flows can be used to pattern or aggregate particles precisely and independently of their size in the tailoring of 2D colloidal crystals.^13^,^14^

Previous work on photoswitchable surfactants that undergo photo-isomerization reactions from *E* to *Z* conformations when irradiated with UV and blue light has concentrated mostly on non-ionic azo surfactants with surface tension changes as high as ∼29 mN m^–1^ upon photoswitching.^15^,^16^ However, information on dynamic changes at interfaces like surface excess and structural information on the molecular level have not been obtained in detail, yet. That is particularly true for water-soluble photoswitches that have much greater potential to serve as building blocks for responsive as well as active colloids and interfaces. To study the underlying mechanism, kinetically resolved measurements at interfaces far outside local^17^ or global equilibria are required. In addition, for most azobenzene derivatives switching is incomplete due to a large spectral overlap of both isomers. Recently, Stricker *et al.*^18^,^19^ showed the potential of arylazopyrazoles (AAPs) as a new class of photoswitches in aqueous solutions, which exhibit optical properties superior to azobenzene derivatives and which were first introduced by Weston *et al.*^20^ Particularly, the smaller spectral overlap^18^ of *E* and *Z* isomers, as well as a more favourable photostationary state, which allows switching of >90% of the molecules, makes AAP derivatives highly interesting as molecular building blocks for responsive air–water interfaces.^19^,^20^

**Fig. 1:**
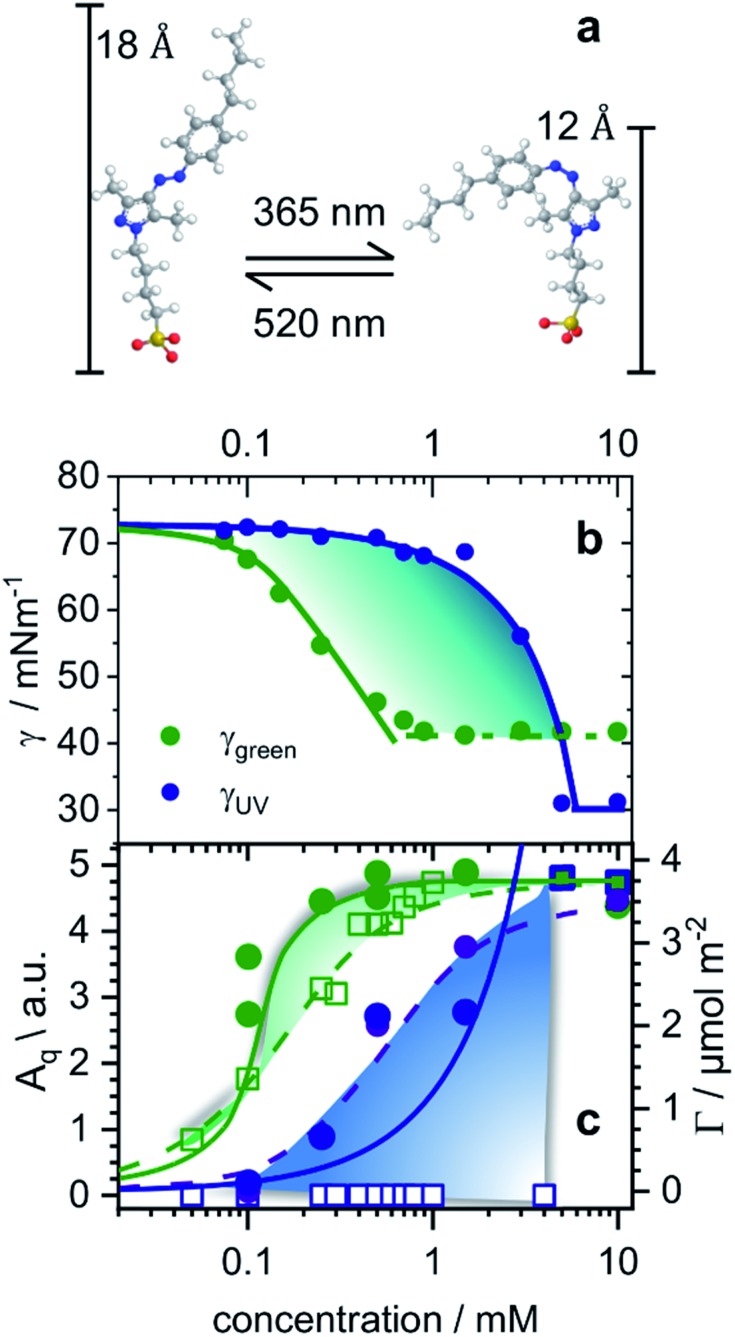
(a) Structures of butyl-AAP-C_4_S surfactants without Na^+^ counterions in *E* (520 nm green light) and *Z* (365 nm UV iight) conformations. (b) Equilibrium surface tension isotherms of butyl-AAP-C_4_S surfactants in the *E* (green symbols) and *Z* state (blue symbols) and isotherms (solid lines) from the Frumkin model using the maximum surface excess *Γ*_max_ from NR. (c) Surface excess *Γ* from the low-*Q* analysis method of NR (see Experimental details) of butyl-AAP-C_4_S surfactants at the air–water interface in equilibrium. Here green and blue circles indicate samples irradiated with green and UV light with the surfactants in the *Z* and *E* states, respectively. (c) The SFG amplitudes *A*_q_ ∝ *Γ*〈*β*_q_〉 from symmetric S–O stretching vibrations of the surfactants in the from symmetric S–O stretching vibrations of the surfactants in the *E* (green squares) state and the *Z* (blue squares) state. Solid lines in (c) show the surface excess that is taken from the Frumkin isotherm fitted to the surface tension in (b). Dashed lines guide the eye, while colour shaded areas indicate the relative difference between *Γ* from NR and the SFG amplitude *A*_q_ ∝ *Γ*〈*β*_q_〉..

In this work, we have studied a newly designed AAP derivative – sodium *n*-butyl-arylazopyrazole butyl sulfonate (butyl-AAP-C_4_S, Fig. 1a) – that is shown to achieve massive changes in the physicochemical properties at the air–water interface which are attributable to an unexpected monolayer-to-bilayer transition. This transition is unique for the molecule we describe in this paper, which can be used to explain the superior performance of this new water soluble butyl-AAP-C_4_S photoswitch.

A prerequisite to unravel the monolayer-to-bilayer transition is a structural investigation of equilibrium and non-equilibrium properties that have not been discussed in detail so far. In fact, for the latter we combine the results of vibrational sum-frequency generation (SFG) spectroscopy, surface tensiometry and neutron reflectometry (NR) where NR yields a model-free quantification of the surface excess and SFG reveals structural aspects of the interface. Both SFG and NR are inherently interface specific methods where the SFG amplitude *A*_q_ ∝ *Γ*〈*β*_q_〉 of a vibrational band is a function of both the surface excess of a vibrational band is a function of both the surface excess *Γ* and the orientational average and the orientational average 〈⋯〉 of the molecular hyperpolarizability ··· and the orientational average 〈⋯〉 of the molecular hyperpolarizability of the molecular hyperpolarizability *β*_q_, while fitted models of the NR data provide the density profile of molecules (or parts of molecules) normal to an interface (see Experimental details). Therefore, a comparison of the two techniques can reveal information about changes in the orientation, structure and coverage of interfacial molecules. Using this combined approach, we first quantify changes in surface tension, surface excess and molecular order to examine the performance of the new surfactants. Finally, the experiments are complemented by two experimental demonstrations of potential applications, which show that the unprecedented monolayer-to-bilayer transition of butyl-AAP-C_4_S surfactants at the air–water interface can be exploited for applications where the surfactant serves as a versatile building block for highly photo-responsive foams and light-actuated particle motion.

## Results and discussion

The equilibrium surface tension of butyl-AAP-C_4_S photoswitchable surfactants as a function of bulk concentration is shown in Fig. 1b for irradiation with 520 nm green and 365 nm UV light. Green and UV light can cause *E* to *Z* isomerization reactions of the amphiphiles, which we have confirmed by ^1^H high-resolution magic-angle spinning (HR-MAS) NMR spectroscopy (ESI†). In particular, ^1^H HR-MAS NMR demonstrates an extremely favorable photostationary state (PSS) with >99% *E* to *Z* switching and complete recovery of the *E* isomer at 6 mM (see Fig. S1†). This is an outstanding performance as other AAP and azo moieties have been reported to have a PSS of >98% (ref. 18 and 20) and <80%.^21^ For <5 mM butyl-AAP-C_4_S, the equilibrium surface tension of the air–water interface is systematically lower when the samples are in the *E* state compared to the *Z* state (Fig. 1b), while for >5 mM the surface tension for the surfactants in the *E* state reaches a plateau at a higher value.

These two differences can be attributed to the different critical micelle concentrations (CMCs) of the butyl-AAP-C_4_S surfactants in the bulk solution and the different equilibrium constants for ad/desorption, respectively. The CMCs are 0.7 and 5 mM for the surfactants having *E* and *Z* conformations, respectively, and can be directly inferred from the kinks in the isotherms of Fig. 1b. From this analysis, we can conclude the relative surface activity of the *E* and *Z* isomers, with the *E* isomer being more surface active. The surface tension change Δ*γ* upon photoswitching is as high as 27 mN m^–1^ for 0.5 mM butyl-AAP-C_4_S and is, therefore, much higher than those of the previously reported cationic amphiphiles, with a difference of ∼20 mN m^–1^.^7^,^8^,^15^ This performance of the butyl-AAP-C_4_S surfactant is close to the 29 mN m^–1^ of nonionic surfactants reported by Shang *et al.*^15^ The fact that this performance is achieved for an ionic surfactant is remarkable as small changes in surface coverage and charge density can easily destabilize these systems, as is demonstrated below.

To gain detailed information about the structural changes of butyl-AAP-C_4_S photoswitches at the air–water interface, we have performed experiments with NR and SFG spectroscopy. In Fig. 1c, the surface excess *Γ* (the number of molecules per surface area) of butyl-AAP-C_4_S surfactants from the low-*Q* analysis of NR as a function of the bulk concentration is shown for surfactants in *E* and *Z* conformations, when the samples were irradiated with green und UV light, respectively. Note that *Q* is the momentum transfer normal to the interface and that 1
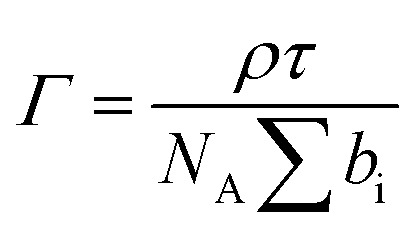
can be calculated in an NR experiment from the scattering length density *ρ*, the fitted layer thickness *τ*, the scattering length *b* of each atom i in each interfacial surfactant molecule, and the Avogadro number *N*_A_. More information on this new model-free implementation of the technique can be found in the experimental section and in a recent review.^22^ From a close analysis of the results in Fig. 1c, the largest change in surface excess *Γ* from one equilibrium state to the other (*E* to *Z*) occurs for a concentration of 0.25 mM butyl-AAP-C_4_S with a surface excess of 3.60 μmol m^–2^ when the surfactants were in the *E* state and 0.66 μmol m^–2^ for the corresponding *Z* state. In order to analyze the data in Fig. 1b in more detail and to compare them with the results in Fig. 1c, we have fitted the surface tension isotherms using a Frumkin model2
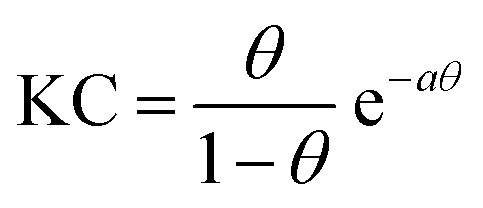
which is typically assumed for the monolayer adsorption of simple surfactants.^23^,^24^ Fits were performed assuming the equation of state of singly charged surfactants

3 *γ*(*c*) = *γ*_0_ + 2*Γ*_max_*RT*[ln(1 – *θ*) + *aθ*^2^]

where *K* is the equilibrium constant for adsorption and desorption of surfactants at the air–water interface, *C* the surfactant bulk concentration, *a* the interaction parameter, *Γ*_max_ the maximum surface excess of the monolayer at concentrations larger than the bulk CMC and *γ*_0_ the surface tension of the neat interface without the presence of surfactants. In addition, *θ* = *Γ*/*Γ*_max_ is the surface coverage, which equals 1 when a monolayer with *Γ*_max_ has been formed.^24^ For the fitting procedure, we have used *Γ*_max_ from our NR experiments (Fig. 1c) as input parameters for the fits. This approach gives good agreement with the experimentally observed equilibrium surface tension *γ* when *a* and *K* were allowed to vary freely in the fitting procedures. The values of *a* are relatively large at 1.6 possibly due to high π-stacking of interfacial surfactants.

Comparing the model free surface excess (NR) with the one from the Frumkin model, we find excellent agreement in case where the surfactants were in the *E* state. This can be directly inferred from the excellent overlap of the surface excess (solid green line in Fig. 1c) taken from the Frumkin fit in Fig. 1b, with the NR data shown in Fig. 1c (green circles). We can also notice for the surfactants being in the *E* conformation that the SFG amplitudes *A*_q_ ∝ *Γ*〈*β*_q_〉 and the surface excess and the surface excess *Γ* ovelaps only at the limiting surface excess, which is reached at or above the CMC of surfactants. Below the CMC, the mismatch between *Γ* and *A*_q_ ∝ *Γ*〈*β*_q_〉 as indicated by the colour-shaded areas in as indicated by the colour-shaded areas in Fig. 1c must be associated with the changes in the orientational average of the hyperpolarizability must be associated with the changes in the orientational average of the hyperpolarizability 〈*β*_q_〉. Thus, the molecular order of the interfacial layer increases with increasing concentration and is likely caused by attractive lateral interactions within the close-packed interfacial layer of surfactants.. Thus, the molecular order of the interfacial layer increases with increasing concentration and is likely caused by attractive lateral interactions within the close-packed interfacial layer of surfactants.

Although modelling of the surface tension of the surfactants in the *E* conformation clearly reproduces the surface excess *Γ* as seen from NR, the same modelling of the results from samples in the *Z* state clearly fails to reproduce the NR data (Fig. 1c), even though the changes in surface tension (Fig. 1b) between the model fit and experimentally observed surface tensions are in good agreement. Application of the Frumkin model, therefore, appears not to be valid for butyl-AAP-C_4_S surfactants in the *Z* conformation. Furthermore, the difference between the surface excess *Γ* and the SFG amplitude *A*_q_ ∝ *Γ*〈*β*_q_〉 of molecules in the of molecules in the *Z* conformation is extreme for concentrations below the CMC (see the blue shaded area in Fig. 1c) because the SFG amplitude is negligible, while the surface excess is still quite substantial. This large difference for concentrations <5 mM must be directly related to the orientational average ) because the SFG amplitude is negligible, while the surface excess is still quite substantial. This large difference for concentrations <5 mM must be directly related to the orientational average 〈*β*_q_〉 with the interfacial surfactants realizing structures with local centrosymmetry which cancel the SFG signals due to symmetry reasons (〈 with the interfacial surfactants realizing structures with local centrosymmetry which cancel the SFG signals due to symmetry reasons (〉 with the interfacial surfactants realizing structures with local centrosymmetry which cancel the SFG signals due to symmetry reasons (〈*β*_q_〉 = 0). This is clearly different for concentrations >5 mM, which is the bulk CMC when the molecules are in the = 0). This is clearly different for concentrations >5 mM, which is the bulk CMC when the molecules are in the *Z* state and is discussed in detail below.

**Fig. 2:**
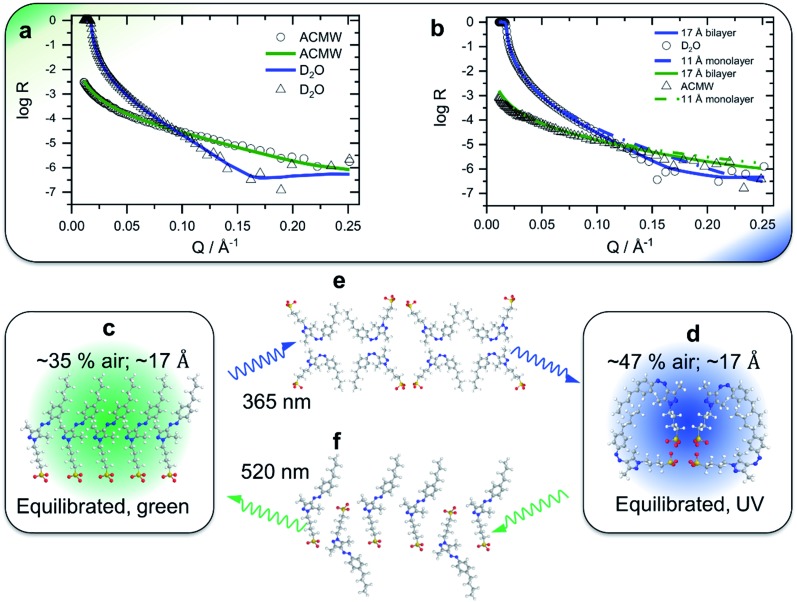
Neutron reflectivity profiles (data points) and optimized model fits (solid lines) of the 0.5 mM partially deuterated butyl-AAP-C_4_S surfactant at the air–D_2_O interface for green (a) and UV (b) irradiation; in the latter case, simulated (non-optimized) fits of a monolayer with a thickness of 11 Å are also shown (dashed-dotted lines) to demonstrate that the presence of a monolayer of surfactant molecules in their *Z* conformation is not supported by the experimental data. Fitting results for other NR contrasts are shown in the ESI.† Schematics of a monolayer-to-bilayer transition of butyl-AAP-C_4_S surfactants at the air–water interface: (c and b) structures for green and UV irradiation, respectively, when the air–water interface is in thermal equilibrium; (e and f) show suggested transition structures that are formed immediately after the *E* to *Z* and *Z* to *E* photo-isomerization reactions, respectively. Note that the presented schematics show possible structures that may be formed at the interface in spite of the simplification of representing quasi-three dimensional structures in two dimensions. We also point out that the sulfonate head groups are likely not in the same plane for electrostatic reasons. Solvating water molecules and counterions are not shown for clarity.

In order to gain insight into the apparent interfacial rearrangements caused by photo-isomerization, we have characterized the structures that self-assemble at the interface in the two equilibrium states (*Z vs. E*) using NR. For that, the NR data were recorded in 4 isotopic contrasts using partially deuterated and fully hydrogenous surfactants in both D_2_O and air contrast matched water (ACMW), which is 8.1% by volume D_2_O in H_2_O, over the full *Q*-range. These data were simultaneously fitted for each system to a common structural model of stratified layers normal to the interface separated by capillary wave roughness (details are in the Experimental part and in the ESI†). From NR it is found that a layer thickness of 17 Å is created for both *E* and *Z* conformations with the molecules adopting a volume fraction of 65% in the former case and 53% in the latter case (Fig. 2a and b). To place these results in the context of the size of the molecule (Fig. 1a), it may be noted that the extended length of the molecule is ≈18 Å when the butyl-AAP-C_4_S molecule is in the *E* conformation, which reduces to ≈12 Å in the *Z* conformation. Consequently, the modeled NR data indicate that there is a relatively dense monolayer of butyl-AAP-C_4_S surfactants in the *E* conformation and a more hydrated and straddled (or intercalated) bilayer when the surfactants are in the *Z* state, which are schematically illustrated in Fig. 2c and d, respectively. In the latter case, the prevailing centrosymmetric environment can explain the complete loss of the SFG signal for surfactant concentrations <5 mM, when they are switched from the *E* to the *Z* state (Fig. 1c). This is clearly different at concentrations >5 mM, where no change in the SFG amplitude, surface excess and layer thickness (ESI, Fig. S6†) as a function of light irradiation is observed. At concentrations >5 mM, the close-packed interfacial layer of surfactants is likely to be sterically arrested in the *E* state due to strong lateral interactions resulting in a loss of its responsiveness to the light stimulus. In fact, this is similar to photo-switchable self-assembled monolayers which also lose their light-responsive function, when their packing becomes too dense.^25^

At this point, we need to stress that the presented structures are consistent with our experimental observations, but they are also a highly schematic representation of the interfacial structure. A more detailed resolution of the specific molecular configurations is, however, outside the scope of this study.

The surprising monolayer-to-bilayer transition for an amphiphilic photoswitch is unprecedented and in fact can explain the origin of the unique performance of this surfactant in the applications we present below. We attribute this unexpected result to the bipolar character of *Z*-butyl-AAP-C_4_S, which is caused by the exposed polar azo group at one end of the molecule and the sulfonate head group at the other end, resulting in the observed bilayer structure.

This intriguing interfacial rearrangement of butyl-AAP-C_4_S upon photoswitching raises the question what actually happens during the conversion of one interfacial structure to the other. As such, we sought to resolve the photoswitching mechanism in real time using the same powerful set of techniques: surface tensiometry, SFG spectroscopy (S–O amplitudes) and the low-*Q* analysis method of NR (model-free surface excess).

**Fig. 3:**
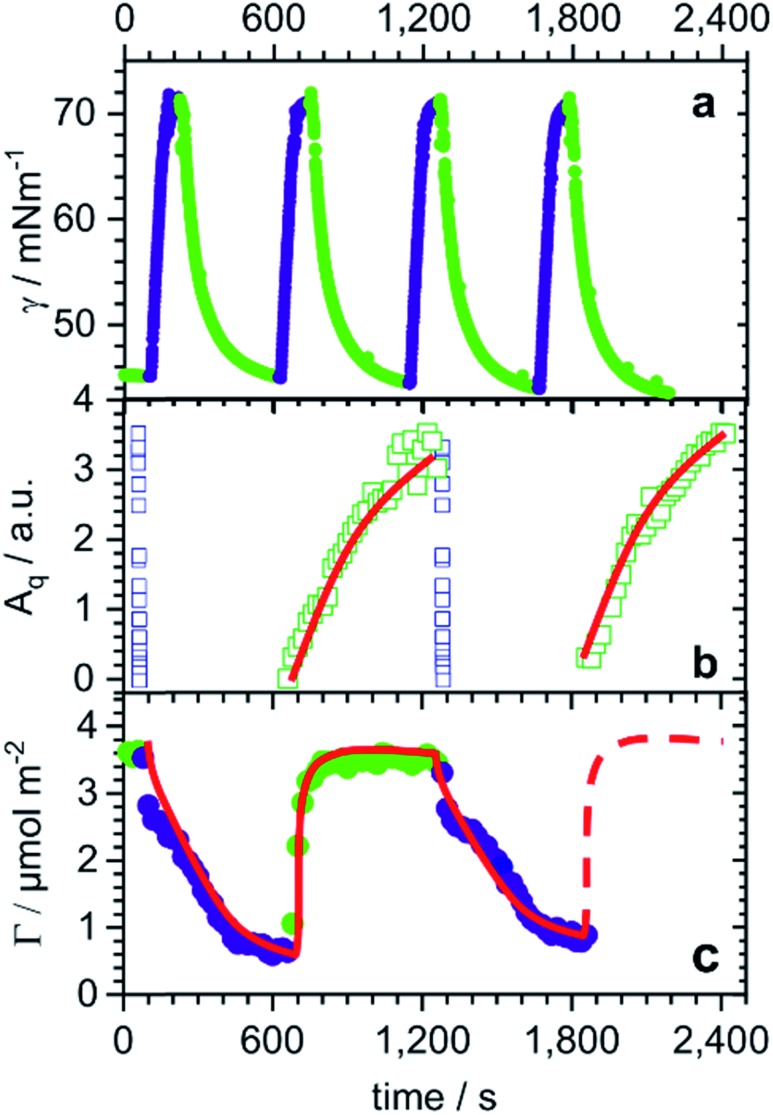
*E*/*Z* switching kinetics for changes in irradiation from 520 nm (green symbols) to 365 nm (dark blue symbols) light and *vice versa*: (a) dynamic surface tension *γ*(*t*) of 0.5 mM butyl-AAP-C_4_S, (b) time-resolved S–O amplitudes of 0.25 mM butyl-AAP-C_4_S from the kinetic SFG spectra shown in the ESI,† and (c) changes in surface excess *Γ* of 0.25 mM butyl-AAP-C_4_S as measured by the low-*Q* analysis method of NR (see Experimental section). Strikingly, timescales of changes in the surface excess are almost inverted with respect to the data from the other two techniques.

During four cycles of photoswitching for 0.5 mM butyl-AAP-C_4_S (chosen due to the largest Δ*γ*; Fig. 1b), a fast increase in the surface tension is observed for *E* to *Z* transitions, while the reduction in surface tension caused by the transition from the equilibrated *Z* to the *E* state is much slower (Fig. 3a). Furthermore, during two cycles of photoswitching for 0.25 mM butyl-AAP-C_4_S (chosen due to the largest Δ*Γ*; Fig. 1c), there is a complete loss in the SFG signal for switching from the *E* to the *Z* state on a time scale of just a few seconds (Fig. 3b), while the reappearance of the SFG signals by switching from the *Z* to the *E* conformation under constant green irradiation takes several minutes. The relative time scales of the response of the interfacial properties to the light stimulus are therefore qualitatively consistent for SFG spectroscopy and surface tensiometry. On the other hand, the kinetic changes in the surface excess *Γ*(*t*) in an equivalent NR experiment are strikingly different. In this case, there is a slower decrease in the surface excess for *E* to *Z* (>10 min) transitions than the corresponding increase for switching from the *Z* to the *E* state (∼1 min). We will in the following rationalize these different responses in the context of the monolayer/bilayer transition elucidated above in order to formulate an interfacial mechanism during photoswitching.

In switching from a relatively dense monolayer with the surfactants in the *E* conformation to a more hydrated bilayer with the surfactants in the *Z* state, a fast loss of the molecular order (SFG) and an increase in surface tension are accompanied by a slow reduction in surface excess (NR). We infer that the monolayer-to-bilayer transition occurs quickly after the transformation from *E* to the sterically more demanding *Z* conformation, which can be interpreted in terms of a rearrangement of half of the surfactant molecules from the monolayer to adopt positions in the lower leaflet of the bilayer. The rearrangement further leads to a molecular configuration having inversion symmetry that annuls the SFG signals with a lower surface coverage, hence, resulting in a sharp increase in surface tension (schematic in Fig. 2c). As a bilayer pair of *Z* surfactant molecules is less amphiphilic (and thereby less surface active) than a single *E* isomer in the monolayer, molecules desorb from the interface, lowering the surface excess slowly without significant further changes in the molecular order or surface tension.

Conversely, in switching from the bilayer (*Z* conformation) to monolayer (*E* conformation), a fast increase in surface excess (NR) is followed by a slower gain in the molecular order (SFG) and a reduction in surface tension. In this case, we infer that the adsorption of additional surfactants from the sub-surface to the interface is fast as the *E* isomer is more surface active than the *Z* isomer (schematic in Fig. 2d). However, the transition to a complete monolayer, which results in a loss of the inversion symmetry giving rise to the SFG signal and reduces the surface tension, is relatively slow.

This interfacial picture is similar to other self-assembly processes like the formation of self-assembled monolayers at solid surfaces, where the close to maximum surface excess is established relatively fast but the assembly into the final highly ordered structures takes place on time scales of many hours.^26^,^27^ Analogous effects at fluid interfaces have so far not been reported, and indeed resolution of a structural mechanism of surfactant photoswitching at the air–water interface is without precedent.

**Fig. 4:**
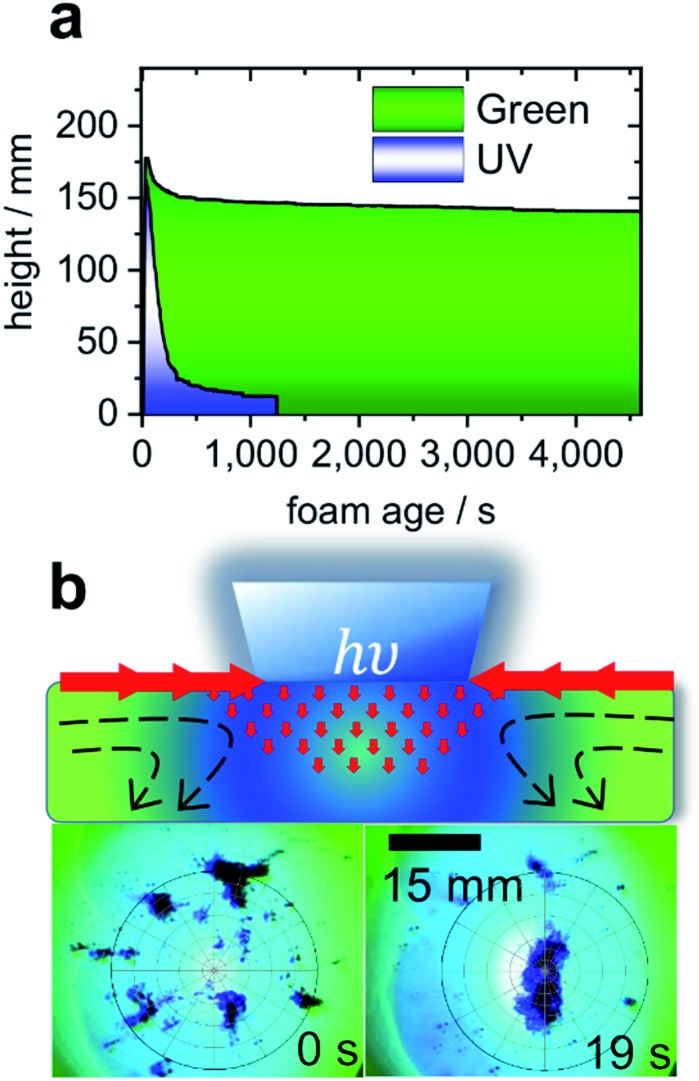
(a) Foam height as a function of foam age for solutions with 0.5 mM butyl-AAP-C_4_S photoswitches. Measurements under 365 nm UV and 520 nm green light irradiation are indicated by blue and green colours. (b) Schematic presentation of light-actuated Marangoni flow of surfactants (molecules not shown) caused by local surface tension gradients when the AAP modified air–water interface is irradiated locally with UV light. In addition, experiments where carbon particles are spread at the air–water interface from 0.5 mM butyl-AAP-C_4_S and the sample was irradiated locally with 365 nm UV light but globally with 520 nm are shown. Full video sequences are available online in the ESI.†

Turning to the application of butyl-AAP-C_4_S, Fig. 4a presents the results for aqueous foams prepared from 0.5 mM butyl-AAP-C_4_S (maximum Δ*γ* upon photoswitching; Fig. 1b). The foam lifetime in terms of decreasing foam height was ≫1 h when irradiated with green light, which is dramatically reduced to ∼150 s when the foam is irradiated with UV light. Clearly, the above discussed changes at the molecular level of the air–water interface have also dramatic consequences at a macroscopic level. Furthermore, the observed foam lifetime for green irradiation (*E* conformation) is much higher than that observed previously for other photo-surfactants, where the process has been shown to take several minutes.^9^,^10^

Aqueous foams are inherently interface controlled, where the macroscopic properties can be tailored at the molecular level through structure–property relations.^2^,^28^ Since the surface excess of an ionic surfactant such as butyl-AAP-C_4_S determines the interfacial charging state, the latter is reduced when the samples are irradiated with UV light. This reduction in surface charge density leads to a considerable reduction in the electrostatic disjoining pressure Π(*h*), which results in coalescence and bursting of foam bubbles.^29^

In the case of AAP stabilized foams, the electrostatic disjoining pressure is the main driving force in foam stability,^2^ and clearly the use of butyl-AAP-C_4_S surfactants can render aqueous foam even more responsive to light irradiation. Indeed, the above described massive and fast light-induced changes at the air–water interface have potential to collapse foam after its desired application, enabling an easy foam removal and recycling of its ingredients in an environmentally friendly way without using antifoaming agents.

Another possibility to exploit the potential of butyl-AAP-C_4_S *via E*/*Z* photo-isomerization in applications involves Marangoni flows^4^,^30^ at the air–water interface, an effect that can be exploited to move micro- and nanoparticles into ordered structures at a fluid interface in the formation of colloidal crystals.^13^ A proof of this concept is shown in Fig. 4b, where >100 μm carbon particles were spread at the air–water interface, aggregated, and moved using light-actuated Marangoni flows of butyl-AAP-C_4_S during photoswitching. Due to the massive and fast changes in the interfacial structure, surface excess, and surface tension upon photoswitching, the visible Marangoni effects where interfacial surfactants migrate along existing surface tension gradients extended over several millimetres away from the UV beam, and the particles were dragged along the surfactant flow field (see ESI videos online†).

In comparison with the results of previous studies,^4^,^13^ the resulting forces produced for this system were quite remarkable as agglomerates of several millimetres were transported over distances >10 mm remotely by photoswitching on demand (see ESI videos online†).

## Conclusions

Air–water interfaces modified with a new photoswitchable surfactant, butyl-AAP-C_4_S, have been studied using surface tensiometry, vibrational sum-frequency generation spectroscopy and neutron reflectometry. Using this powerful approach, first we demonstrate that dramatic changes in the surface tension and surface excess can be caused by *E* to *Z* photo-isomerization of the surfactant. The experimental evidence from complementary methods points to an unexpected monolayer-to-bilayer transition of the surfactant at the air–water interface, which is attributed to the unique molecular structure of butyl-AAP-C_4_S whose amphiphilicity varies in the two isomers. The transition structures adopted at the interface during photoswitching are proposed in an unprecedented resolution of the structural mechanism and are key to the superior performance of this new surfactant. Thus, for interface modifications, butyl-AAP-C_4_S seems to be the perfect photoswitch because of the unprecedented monolayer-to-bilayer transition which we have shown for the first time.

The performance of the new surfactant based on the underlying monolayer to bilayer transition was demonstrated in two applications: (i) highly photo-responsive aqueous foams where the foam stability can be tuned from hours to minutes using green and UV light irradiation, respectively, and (ii) light-triggered Marangoni flows where a localized light stimulus resulted in massive and long-range particle movements. Clearly, these properties of butyl-AAP-C_4_S, not only at the molecular level but also at mesoscopic and macroscopic length scales, show great potential to be exploited as building blocks of adaptive materials in the future.

## Experimental section

### Vibrational sum-frequency generation (SFG) spectroscopy

SFG spectroscopy is an inherent interface specific method for materials with inversion symmetry and is based on a second-order nonlinear optical process. In a simplified form, the SFG intensity can be written as follows.^31^4
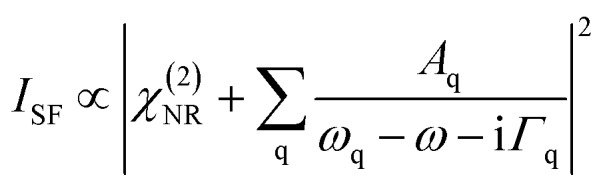
where, *χ*(2)NR, *Γ*_q_, and *ω*_q_ are the nonresonant contribution to the second-order susceptibility, the Lorentzian linewidth, and the resonance frequency of the q^th^ vibrational mode, respectively. In addition, *A*_q_ ∝ *Γ*〈*μ*_q_*α*_q_〉 = = *Γ*〈*β*_q_〉 is the amplitude of the mode q, and is directly related to the surface excess is the amplitude of the mode q, and is directly related to the surface excess *Γ* of amphiphilic molecules as well as to the orientational average of both the dynamic dipole moment *μ*_q_ of molecules and their Raman polarizability *α*_q_.^32^,^33^ This orientational average has far reaching implications as only molecules or vibrational modes in non-centrosymmetric environments can contribute to SFG signals. For this reason, SFG is inherently interface specific for materials with centrosymmetry such as liquids and gases. At interfaces, like the air–water interface in this study, the centrosymmetry of the bulk is necessarily broken and both the polarity as well as the magnitude of the SFG amplitude of a vibrational band are determined by the net molecular orientation of interfacial species. However, molecular structures at interfaces can also be centrosymmetric, which would render such structures ‘silent’ in the SFG spectra. As an example the reader is referred to close-packed self-assembled monolayers^27^,^32^ with a long alkyl chain in the all-*trans* conformation. Here, the methylene groups exhibit local centrosymmetry and their symmetric stretching mode does not contribute to the SFG spectra.^27^ Note that the above expression can also be used to fit the experimental SFG spectra with model functions, *e.g.*, using Lorentzian line shapes as in eqn (4). Indeed, this approach was used in order to obtain more quantitative information from the SFG spectra. If not noted otherwise the SFG spectra were recorded in SSP polarizations. Details of the SFG spectrometer which was used in this study can be found elsewhere^34^ and in the ESI.†

### Neutron reflectometry (NR)

Two different implementations of NR were used to resolve either the dynamic surface excess or the interfacial structure of butyl-AAP-C_4_S at the air–water interface. The FIGARO instrument at the Institut Laue-Langevin (Grenoble, France) was used.^35^ The time-of-flight instrument was used with a broad chopper pair defining the neutron pulses to exploit high flux at the expense of loose resolution of 8% (FWHM) in the momentum transfer, *Q*, given by5
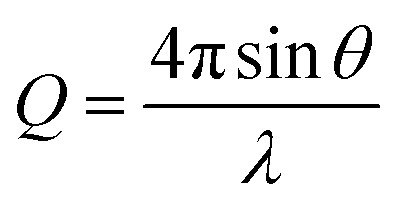
where *θ* is the incident angle and *λ* is the wavelength.

In the first implementation of NR, the recently established method of low-*Q* analysis was used,^36^ where data were recorded only at *θ* = 0.62° and in the range of *λ* = 2–16 Å, and they were reduced only in the range *λ* = 4.5–12 Å to give the *Q*-range 0.01–0.03 Å^–1^. This approach desensitized the measurements to the interfacial structure. This method has been used to good effect in the last few years to resolve kinetic and dynamic processes at fluid interfaces.^22^ The measurements were carried out on normal hydrogenous and partially deuterated surfactants on a subphase of ACMW, which has zero scattering length density, hence, the specular reflection is dominated by the signal from the adsorbed surfactant. As a result, the low-*Q* range and the nature of the measurements, a single mixed interfacial layer of surfactant tails and headgroups was used to fit the data. The surface excess *Γ* is calculated from eqn (1) as stated above. The background was not subtracted from the data, and the value of the background used in the model was 3.48 × 10^–5^ as derived from measurements of pure ACMW.

In the second implementation of NR, the data were recorded at *θ* = 0.62° and 3.8° in the range of *λ* = 2–30 Å. The measurements were carried out both on hydrogenous and partially deuterated surfactants on subphases of both ACMW and D_2_O. A common structural model of stratified layers normal to the interface was applied simultaneously to the data recorded in all four isotopic contrasts. The general modelling principles outlined in a recent paper^37^ were followed. Here, an upper layer of surfactant chains had a fitted thickness, a lower layer of surfactant headgroups had its solvation constrained so that the number of chains and headgroups was equal, and capillary wave roughness was calculated from the surface tension. As the molecules in this study include double bonds that result in constrained molecular orientations, the volume fraction of the layer of chains was fitted rather than being constrained to 1. For this measurement, the background was subtracted from the data. In the experiment, samples were measured in sequence on the standard 6-position sealed adsorption trough sample changer of the instrument. The lids of the trough were specially adapted to hold green and UV LEDs positioned 2.2 cm above the center of each liquid surface. The data were analysed using Motofit.^38^

## Conflicts of interest

There are no conflicts to declare.

## Supplementary Material

Supplementary information

Supplementary movie

Supplementary movie

## References

[cit1] Zhao T., Zhu X., Zhang L., Cang H., Zhang X., Li C., Wei H., Ma N. (2018). ACS Appl. Mater. Interfaces.

[cit2] Schnurbus M., Stricker L., Ravoo B. J., Braunschweig B. (2018). Langmuir.

[cit3] Weißenborn E., Braunschweig B. (2019). Soft Matter.

[cit4] Lv C., Varanakkottu S. N., Baier T., Hardt S. (2018). Nano Lett..

[cit5] (c) Takashima Y., Hatanaka S., Otsubo M., Nakahata M., Kakuta T., Hashidzume A., Yamaguchi H. and Harada A., Nat. Commun., 2012, 3, 1270. 23232400 10.1038/ncomms2280 PMC3535346

[cit6] Wu Z., Xue R., Xie M., Wang X., Liu Z., Drechsler M., Huang J., Yan Y. (2018). J. Phys. Chem. Lett..

[cit7] Chevallier E., Mamane A., Stone H. A., Tribet C., Lequeux F., Monteux C. (2011). Soft Matter.

[cit8] Fei L., Ge F., Yin Y., Wang C. (2019). Colloids Surf., A.

[cit9] Chen S., Zhang Y., Chen K., Yin Y., Wang C. (2017). ACS Appl. Mater. Interfaces.

[cit10] Chevallier E., Monteux C., Lequeux F., Tribet C. (2012). Langmuir.

[cit11] Cheng J., Štacko P., Rudolf P., Gengler R. Y. N., Feringa B. L. (2017). Angew. Chem., Int. Ed..

[cit12] Carl A., von Klitzing R. (2011). Angew. Chem., Int. Ed..

[cit13] Vialetto J., Anyfantakis M., Rudiuk S., Morel M., Baigl D. (2019). Angew. Chem., Int. Ed..

[cit14] Yakovlev E. V., Komarov K. A., Zaytsev K. I., Kryuchkov N. P., Koshelev K. I., Zotov A. K., Shelestov D. A., Tolstoguzov V. L., Kurlov V. N., Ivlev A. V., Yurchenko S. O. (2017). Sci. Rep..

[cit15] Shang T., Smith K. A., Hatton T. A. (2003). Langmuir.

[cit16] Brown P., Butts C. P., Eastoe J. (2013). Soft Matter.

[cit17] Backus E. H. G., Kuiper J. M., Engberts J. B. F. N., Poolman B., Bonn M. (2011). J. Phys. Chem. B.

[cit18] Stricker L., Böckmann M., Kirse T. M., Doltsinis N. L., Ravoo B. J. (2018). Chem.–Eur. J..

[cit19] Stricker L., Fritz E.-C., Peterlechner M., Doltsinis N. L., Ravoo B. J. (2016). J. Am. Chem. Soc..

[cit20] Weston C. E., Richardson R. D., Haycock P. R., White A. J. P., Fuchter M. J. (2014). J. Am. Chem. Soc..

[cit21] Cicciarelli B. A., Hatton T. A., Smith K. A. (2007). Langmuir.

[cit22] Campbell R. A. (2018). Curr. Opin. Colloid Interface Sci..

[cit23] Kairaliyeva T., Aksenenko E. V., Mucic N., Makievski A. V., Fainerman V. B., Miller R. (2017). J. Surfactants Deterg..

[cit24] Stubenrauch C., Fainerman V. B., Aksenenko E. V., Miller R. (2005). J. Phys. Chem. B.

[cit25] Valley D. T., Onstott M., Malyk S., Benderskii A. V. (2013). Langmuir.

[cit26] Schwartz D. K. (2001). Annu. Rev. Phys. Chem..

[cit27] Meltzer C., Yu H., Peukert W., Braunschweig B. (2018). Phys. Chem. Chem. Phys..

[cit28] Streubel S., Schulze-Zachau F., Weißenborn E., Braunschweig B. (2017). J. Phys. Chem. C.

[cit29] Foam Films and Foams: Fundamentals and Applications, ed. D. Exerowa, G. Gochev, D. Platikanov, L. Liggieri and R. Miller, CRC Press, 2019.

[cit30] Arangalage M., Li X., Lequeux F., Talini L. (2018). Soft Matter.

[cit31] Ohno P. E., Wang H.-F., Geiger F. M. (2017). Nat. Commun..

[cit32] Bain C. D., Davies P. B., Ong T. H., Ward R. N., Brown M. A. (1991). Langmuir.

[cit33] de Aguiar H. B., de Beer A. G. F., Strader M. L., Roke S. (2010). J. Am. Chem. Soc..

[cit34] García Rey N., Weißenborn E., Schulze-Zachau F., Gochev G., Braunschweig B. (2019). J. Phys. Chem. C.

[cit35] Campbell R. A., Wacklin H. P., Sutton I., Cubitt R., Fragneto G. (2011). Eur. Phys. J. Plus.

[cit36] Braun L., Uhlig M., von Klitzing R., Campbell R. A. (2017). Adv. Colloid Interface Sci..

[cit37] Campbell R. A., Saaka Y., Shao Y., Gerelli Y., Cubitt R., Nazaruk E., Matyszewska D., Lawrence M. J. (2018). J. Colloid Interface Sci..

[cit38] Nelson A. (2006). J. Appl. Crystallogr..

