# Supplementary material for: Unexpected monolayer-to-bilayer transition of arylazopyrazole surfactants facilitates superior photo-control of fluid interfaces and colloids
Source: Chem Sci. 2020 Jan 8;11(8):2085–92. doi: 10.1039/c9sc05490a (PMC7059314; doi:10.1039/c9sc05490a)
Supplement: Supplementary file 1 [file SC-011-C9SC05490A-s001.pdf]

Electronic Supplementary Material (ESI) for Chemical Science.
This journal is © The Royal Society of Chemistry 2020

ELECTRONIC SUPPORTING INFORMATION

Unexpected monolayer-to-bilayer transition of arylazopyrazole surfactants facilitates superior photo-control of fluid interfaces and colloids

Christian Honnigfort[1,2], Richard A. Campbell[3], Jörn Droste[1], Philipp Gutfreund[4], Michael Ryan Hansen[1], Bart Jan Ravoo[2,5] and Björn Braunschweig[1,2]*

[1] Institute of Physical Chemistry, Westfälische Wilhelms-Universität Münster, Corrensstraße 28/30, 48149 Münster, Germany
[2] Center for Soft Nanoscience (SoN), Westfälische Wilhelms-Universität Münster, Busso-Peus-Straße 10, 48149 Münster Germany
[3] Division of Pharmacy & Optometry, School of Health Sciences, University of Manchester, Oxford Road, Manchester M13 9PT, United Kingdom
[4] Institut Laue-Langevin, 71 avenue des Martyrs, CS 20156, 38042 Grenoble Cedex 9, France
[5] Organic Chemistry Institute, Westfälische Wilhelms-Universität Münster, Corrensstraße 40, 48149 Münster, Germany
*e-mail: braunschweig@uni-muenster.de.

DOI:

1. **Author Contributions** ... 2
2. **Methods** ... 2
3. **NMR Spectroscopy and Photostationary States** ... 2
4. **Synthesis: General Procedure** ... 3
5. **UV-Vis Spectroscopy** ... 5
6. **Brief Description of the Spectrometer for Sum-Frequency Generation** ... 6
7. **Kinetic SFG Spectra of AAP Photo-Switches** ... 7
8. **SFG Spectra of O-H and C-H stretching bands** ... 7
9. **Fitting of Neutron Reflectometry Data** ... 7
10. **Spectra of LEDs for sample irradiation** ... 12
11. **References** ... 12

ELECTRONIC SUPPORTING INFORMATION

1. Author Contributions

B.B. conceived the project and the scope of the manuscript; C.H. performed and B.J.R. supervised the surfactant synthesis and characterization; C.H. performed and B.B. supervised the tensiometry, vibrational SFG spectroscopy, foam lifetime and Marangoni flow experiments and their analysis; J.D. and M.R.H. performed and analyzed the NMR experiments determined the photostationary state of the bulk solution; R.A.C and B.B. devised the scope of the NR experiments; C.H., R.A.C., P.G. and B.B. performed the NR experiments; C.H. and R.A.C. performed the NR data analysis; C.H., R.A.C. and B.B. primarily wrote the submitted version of the manuscript; all authors revised the submitted version of the manuscript and approved the submission.

2. Methods

Sample Preparation

butyl-AAP-C_4S (Figure 1) surfactants were synthesized and characterized with NMR and MS spectroscopy as explained in detail in the Supplementary Information. The chemicals applied for the synthesis were used without further purification. Solvents were dried before use. Sample solutions were prepared by dilution of a freshly prepared stock solution. The latter was obtained by dissolving the necessary amount of photo surfactants in ultrapure water. Sample solutions were sonicated at room temperature for at least 10 min before use. Ultrapure water was received from a Milli-Q Reference A+ water purification system (Merck, Germany) with a resistance of >18 MΩ·cm and total oxidizable carbon < 4 ppb. Prior use, all glassware was stored in a bath of concentrated sulfuric acid 98 % (Carl Roth, Germany) and Nochromix (Godax Labs, USA) for at least 12 h and rinsed thoroughly with ultrapure water and dried in a stream of argon. All experiments were conducted at 295 K except for NR where the ambient temperature was 299 K.

Sample irradiation

Samples were illuminated with the help of LEDs with 365 nm (Roschwege, Star-UV365-05-00-00) and 520 nm (Roschwege, LSC-G) center wavelength. Light intensity was controlled via variation of the supplied current.

Surface tensiometry

The dynamic changes in surface tension were measured with a pendant drop[1] tensiometer (PAT 1M Sinterface, Germany) where the sample compartment was equipped with OG590 (Schott, Germany) bandpass filters to block wavelengths <590 nm from the tensiometer's light source. This measure prevented unwanted switching of the AAP surfactants due to the instrument itself. The drop shape was analyzed using image analysis and the application of the Young-Laplace equation. Perpendicular to the optical bench of the tensiometer UV (365 nm) and green (520 nm) LEDs were mounted on both sides of the pendant drop within the sample compartment. Analysis of surface tension isotherms was performed using the freely available IsoFit[2] software.

Foam analysis

Foam analysis was done using a dynamic foam analyzer (Krüss DFA100, Germany) that was equipped with a panel of LEDs emitting in the near infrared and a photodiode line detector for measurements of the foam height. In addition, green and UV LEDs were positioned along the foam column in order to achieve a homogeneous irradiation of the foam column.

Marangoni flow experiments

Using a Petri dish with a diameter of 20 mm and 0.5 mM aqueous solutions of butyl-AAP-C_4S, carbon particles were spread at the air-water interface. The setup was homogeneously irradiated with 520 nm green light, while the localized UV light was used to switch the photo surfactants and to generate a surface tension gradient. Videos of the resulting particle motions were taken.

3. NMR Spectroscopy and Photostationary States

The ^1H HR-MAS NMR experiments were conducted on a Bruker Avance NEO (11.74 T, $\nu_L(^1H)$ = 500 MHz) using a 4 mm H/F/X MAS DVT probe equipped with magic-angle gradient coils. All samples were placed in 4.0 mm ZrO_2 HR-MAS rotors with an upper spacer made of Teflon and sealed with a Kel-F cap. The active volume inside the rotor is 50 µL. Adamantane was used as external reference for referencing the chemical shift scale ($\delta(^1H)$ = 1.85 ppm)[3] and optimizing the rf-field strength (ν_{RF} = 62.5 kHz and $\tau_{\pi/2}$ = 4.0 µs). All ^1H HR-MAS NMR spectra were recorded employing 5.0 kHz with a relaxation delay of 30 s. After reaching the spinning rate of 5.0 kHz the sample was left to equilibrate for 5 min. For all irradiation steps two spectra were recorded. First, a single pulse ^1H HR-MAS NMR spectrum was recorded and secondly, a ^1H HR-MAS NMR spectrum where the HDO peak was suppressed using the WATERGATE (W5) sequence[4]. A new shim was setup for all irradiation steps to obtain the best possible ^1H linewidths. The HDO peak of all ^1H NMR spectra was set to the same chemical shift (4.71 ppm) to have identical integration regions.

The ^1H HR-MAS NMR study of the bulk switching properties of butyl-AAP-C_4S was performed on a 6 mM sample dissolved in D_2O. All irradiation experiments were performed ex situ in borosilicate vials using the LED setup described above. Initially, an equilibrated, non-radiated sample was measured as the starting point. Then the sample was irradiated with green light (520 nm, 300 mA, 15 min) to obtain the *E* isomer. Afterwards, the sample was switched to the *Z* isomer using UV light (365 nm, 400 mA, 15 min) and then back to the *E* isomer (520 nm, 300 mA, 15 min) to follow all possible irradiation processes as summarized in Figure S1. Here, the ^1H chemical shift of the aromatic and methyl protons of AAP as well as the protons of the first CH_2 group connected to the AAP nitrogen are clearly sensitive to the isomerization. These protons are colour labelled in Figure S1. The ^1H HR-MAS NMR spectrum of AAP (Figure S1a) after the first irradiation

with green light shows the ^{1}H signals corresponding to the thermodynamic equilibrium for the *E* and *Z* isomer (90:10). After irradiation with UV light the peaks of the *E* isomer disappear completely and only the ^{1}H signals corresponding to the *Z* isomer are visible, i.e., a switching efficiency of >99 % is observed. Additionally, the second switching with green light back to the thermodynamic equilibrium shows a complete recovery of the *E* and Z isomer Figure S1c. Thus, the characterization of the bulk switching properties using ^{1}H HR-MAS NMR shows that the chosen UV wave lengths are sufficient to control the isomerization and that the high switching efficiency of butyl-AAP-C_4S makes it a favourable candidate for this works further investigations.

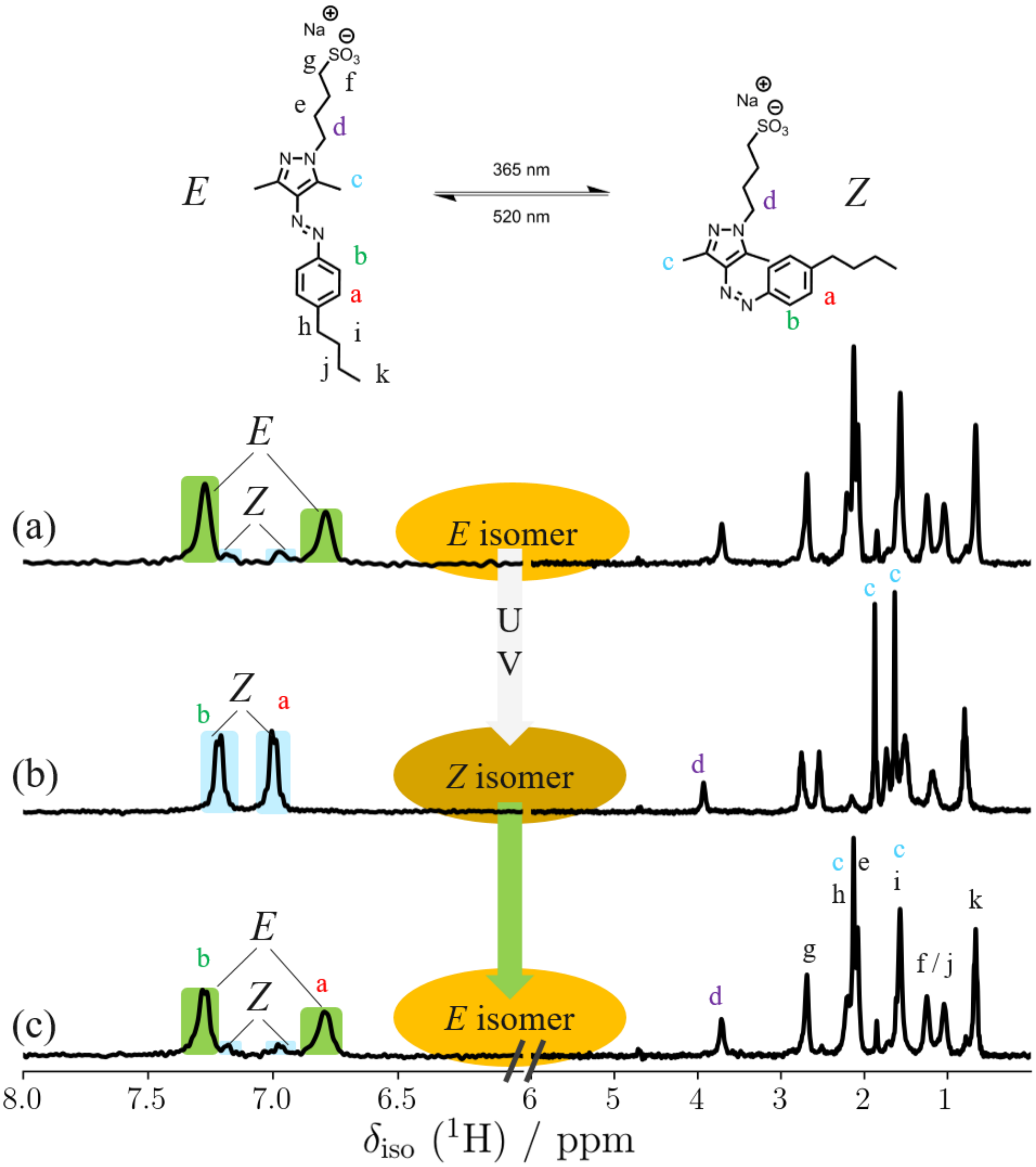

***Figure S 1**: ^{1}H HR-MAS NMR spectra of 6 mM Butyl-AAP-C_4S in D_2O measured at 11.74 T employing a MAS frequency of 5.0 kHz and WATERGATE for water-signal suppression after (a) irradiation with green light (520 nm) (b) switching to the Z isomer with UV light (365 nm), and (c) switching back to the E isomer with green light (520 nm). The chemical structures of the E and Z isomers are shown on top of the figure. The ^{1}H signals affected by the isomerization are colour coded. The asterisk denotes the water signal.*

4. Synthesis: General Procedure

The synthesis of the alkylated AAP was adapted from the literature, according to the procedure published by Weston et al.[5] Chemicals were used as received unless stated otherwise. Hydrogenated and deuterated molecules were synthesized in accordance to the same protocol. NMR Spectra were referenced to the residual solvent peak of *d*-chloroform (99%, Sigma Aldrich) and d_4-methanol (99%, Sigma Aldrich), respectively.

Diketone Precursors

The aniline (1.0 eq.) (97%, Acros Organics, isotopically labeled compound: CDN Isotopes) was dissolved in a mixture of hydrochloric acid (12M, 0.23 mL / mmol) (37%, VWR) and glacial acetic acid (17.4 M, 1.5 mL/mmol) (>99%, VWR). The mixture was cooled to 0°C in an ice

bath. Subsequently sodium nitrite (1.2 eq.) (99.4%, VWR) dissolved in a minimum amount of ultrapure water was added dropwise to the aniline solution. Then the reaction mixture was stirred at 0 °C for 1 hour.

Following this the aforementioned aniline solution was added to 2,4-pentandione (1.3 eq.) (>99%, VWR) and sodium acetate (anhydrous, 3 eq.) (99%, abcr) dissolved in ethanol (1 mL/mmol) (99.8%, Fisher Scientific) and ultrapure water (572 µL/mmol) at room temperature. The mixture was stirred for 1 hour. Vacuum filtration of the yellowish suspension and thorough washing with cold ultrapure water, cold water/ethanol mixture (50: 50), and cold hexane (≥95%, VWR) gave the target compound. The compounds were used without further purification.

3-(2-(4-butylphenyl)hydrazono)pentane-2,4-dione

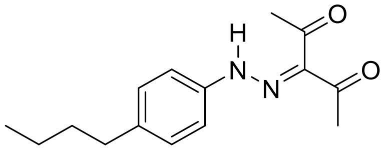

M ($C_{15}H_{20}N_2O_2$): 260.34 g mol-1

Yield: 68% (272 mg, 1.04 mmol) as a bright yellow solid.

^{1}HNMR (300 MHz, $CDCl_3$) δ = 14.81 (s, 1H), 7.32 (d, J = 8.5 Hz, 2H), 7.21 (d, J = 8.5 Hz, 2H), 2.62 (d, J = 7.7 Hz, 2H), 2.59 (s, 4H), 2.48 (s, 3H), 1.65 – 1.54 (m, 2H), 1.35 (h, J = 7.3 Hz, 2H), 0.93 (t, J = 7.3 Hz, 3H) ppm.

MS (ESI, MeOH): (m/z) calculated for $[C_{15}H_{20}N_2O_2Na]^+$: 283.1417 found: 283.1422.

3-(2-(4-(butyl-*d*$_9$)phenyl-2,3,5,6-*d*$_4$)hydrazono)pentane-2,4-dione

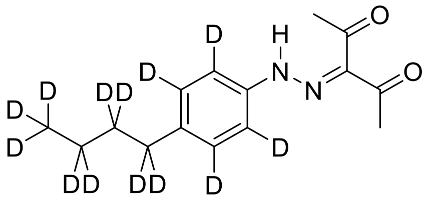

M ($C_{15}H_7 D_{13}N_2O_2$): 273.42 g mol-1

Yield: 87% 362 mg, 1.32 mmol) as a bright yellow solid.

MS (ESI, MeOH): (m/z) calculated for $[C_{15}H_7 D_{13}N_2O_2Na]^+$: 296.2223 found: 296.2229

Arylazopyrazoles

The diketone precursor (1.0 eq.) was dissolved in absolute ethanol (12.5 mL/mmol). Aqueous hydrazine solution (35 wt%, 1 eq) (Sigma Aldrich) was added and the mixture was refluxed for 3 h. Solvent removal *in vacuo* yielded the target compound.

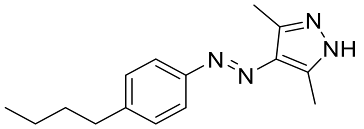

(*E*)-4-((4-butylphenyl)diazenyl)-3,5-dimethyl-1H-pyrazole

M ($C_{15}H_{20}N_4$): 256.35 g mol^{-1}

Yield: 95% 252.3 mg, 0.98 mmol) as an orange solid.

^{1}H-NMR (300 MHz, $CDCl_3$) delta = 9.65 (s, 1H), 7.71 (d, J = 8.4 Hz, 1H), 7.27 (d, J = 8.3 Hz, 1H), 2.67 (t, J = 7.7 Hz, 2H), 2.60 (s, 4H), 2.17 (s, 3H), 1.70 – 1.56 (m, 1H), 1.38 (h, J = 7.3 Hz, 2H), 0.94 (t, J = 7.3 Hz, 2H) ppm.

MS (ESI, MeOH) (m/z) Calc. For $[C_{15}H_{21}N_4]^+$: 257.1761 found:257.1775

(*E*)-4-((4-(butyl-*d*$_9$)phenyl-2,3,5,6-*d*$_4$)diazenyl)-3,5-dimethyl-1H-pyrazole

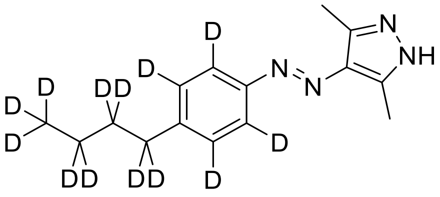

M ($C_{15}H_7 D_{13}N_4$): 269.43 g mol^{-1}

Yield: 97% 160.4 mg, 0.595 mmol) as an orange solid.

MS (ESI, MeOH): (m/z) calculated for $[C_{15}H_7 D_{13}N_4Na]^+$: 270.2577 found: 270.2588

Arylazopyrazole Sodium Sulfonates

This procedure was carried out under an argon atmosphere using standard Schlenk techniques. Sodium hydride (1 eq., 60% dispersion in mineral oil) (Sigma Adrich) was suspended in tetrahydrofuran (5 mL/mmol, dried over molecular sieve) (≥99.5%, VWR). Slowly the arylazopyrazole (1 eq.) was added and stirred for 15 min. Subsequently 1,4-butanesultone (≥99%, Sigma Aldrich) was added and the mixture was refluxed for 24 h. Solvent removal *in vacuo* and purification by column chromatography (silica, ethyl acetate (≥99.8%, Merck)/methanol (≥99%, Fisher Scientific) yielded the desired arylazopyrazole sodium sulfonate.

Sodium (*E*)-4-(4-((4-butylphenyl)diazenyl)-3,5-dimethyl-1H-pyrazol-1-yl)butane-1-sulfonate

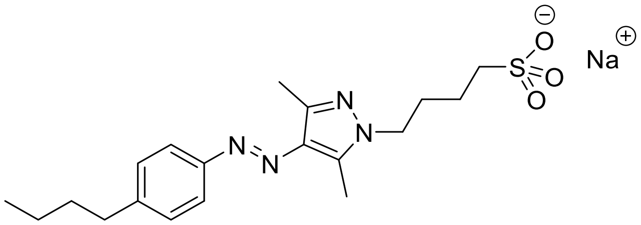

M ($C_{19}H_{27}N_4NaO_3S$): 414.45 g mol^{-1}

Yield: 98%, 404.4 mg, 0.98 mmol) as an orange solid.

^{1}H-NMR (300 MHz, MeOD) δ = 7.69 (d, *J* = 8.3 Hz, 2H), 7.29 (d, *J* = 8.3 Hz, 2H), 4.15 (t, *J* = 7.0 Hz, 2H), 2.85 (t, 2H), 2.68 (t, *J* = 7.7 Hz, 2H), 2.64 (s, 3H), 2.47 (d, *J* = 0.9 Hz, 3H), 2.00 (p, *J* = 7.2 Hz, 2H), 1.82 (p, *J* = 7.7, 7.2 Hz, 2H), 1.65 (p, *J* = 7.5 Hz, 2H), 1.39 (h, *J* = 7.4 Hz, 2H), 0.96 (t, *J* = 7.3 Hz, 3H).

MS (ESI, MeOH) (m/z) Calc. For $[C_{19}H_{27}N_4Na_2O_3S]^+$: 437.1594 found:437.1590.

Sodium (*E*)-4-(4-((4-(butyl-*d*$_9$)phenyl-2,3,5,6-d4)diazenyl)-3,5-dimethyl-1H-pyrazol-1-yl)butane-1-sulfonate

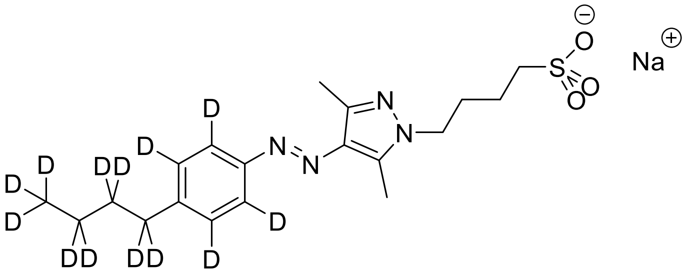

M ($C_{19}H_{14}D_{13}N_4NaO_3S$): 427.58 g mol^{-1}

Yield: 90% 228.8 mg, 0.535 mmol) as an orange solid.

^{1}H-NMR (300 MHz, MeOD) δ = 4.20 – 4.14 (m, 3H), 2.90 (t, J = 7.7 Hz, 2H), 2.65 (s, 3H), 2.49 (s, 3H), 2.04 – 1.96 (m, 2H), 1.96 – 1.75 (m, 2H).

MS (ESI, MeOH): (m/z) calculated for $[C_{19}H_{14}D_{13}N_4O_3S]^-$: 404.2625 found: 406.2643.

5. UV-Vis Spectroscopy

UV-Vis spectra were taken with a Perkin-Elmer Lambda 650 spectrometer using PMMA semi micro cuvettes with 0.1 mM aqueous solutions of the surfactant. LEDs were placed directly above the cuvette and the solution was irradiated for 5 min before spectra were taken.

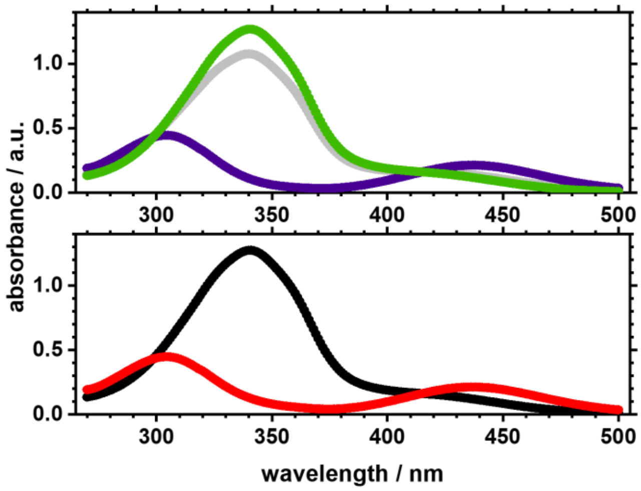

Figure S 2: *Absorption spectra of butyl-AAP-C$_4$S. (a) Under ambient conditions (grey), after irradiation with 520 nm (green) and 365 nm (dark blue), respectively. (b) Spectra recorded of samples equilibrated under 520 nm (black) and 365 nm (red) and subsequent irradiation with 620 nm.*

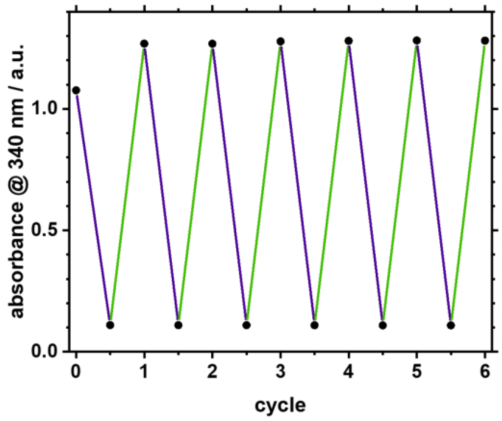

Figure S 3: *Absorbance of a 0.1 mM aqueous solution of butyl-AAP-C$_4$S at a wavelength of 340 nm during 6 continuous cycles of switching. Samples were irradiated for 5 min with 365 nm (dark blue lines) and 520 nm (green lines).*

6. Brief Description of the Spectrometer for Sum-Frequency Generation

Sum-frequency generation (SFG) spectroscopy was performed with a homebuilt spectrometer that is composed of a Solstice Ace chirped pulse amplification system (Spectra Physics, USA) with an overall pulse energy of >7 mJ at 1 kHz and a pulse duration of 70 fs. A 50:50 beam splitter before the internal pulse compressor is used to split the pulse energy in half. Consequently, half of the available pulse energy is compressed to 70 fs and 3.5 mJ in the internal compressor and was used to pump an optical parametric amplifier TOPAS Prime (Light Conversion, Lithuania) and a subsequent unit for non-collinear difference frequency generation (Light Conversion, Lithuania). From the latter, tunable broadband IR pulses with a bandwidth of >300 cm^{-1} were obtained. The remainder of the split beam was compressed externally and filtered with an etalon to achieve time-asymmetric pulses with a duration of 1.2 ps and a spectral bandwidth of 4 cm^{-1} at 804.1 nm center wavelength. Beam blocks in the external compressor were used to remove the side bands after etalon filtering and to clean up the spectrum without compromising pulse shape and beam profile. Both beams were overlapped in time and space at the air-water interface, which was prepared in a quartz glass petri dish and where the sum-frequency was generated and subsequently detected with a combination of a Kymera spectrograph and a Newton EMCCD (both Andor, UK). The sample compartment and the beam path of the broadband IR pulse were purged with dry air using a purge gas generator (CMC Instruments, Germany) in order to avoid IR absorption form the water in ambient air. Further details on the spectrometer setup are presented elsewhere.[6]

7. Kinetic SFG Spectra of AAP Photo-Switches

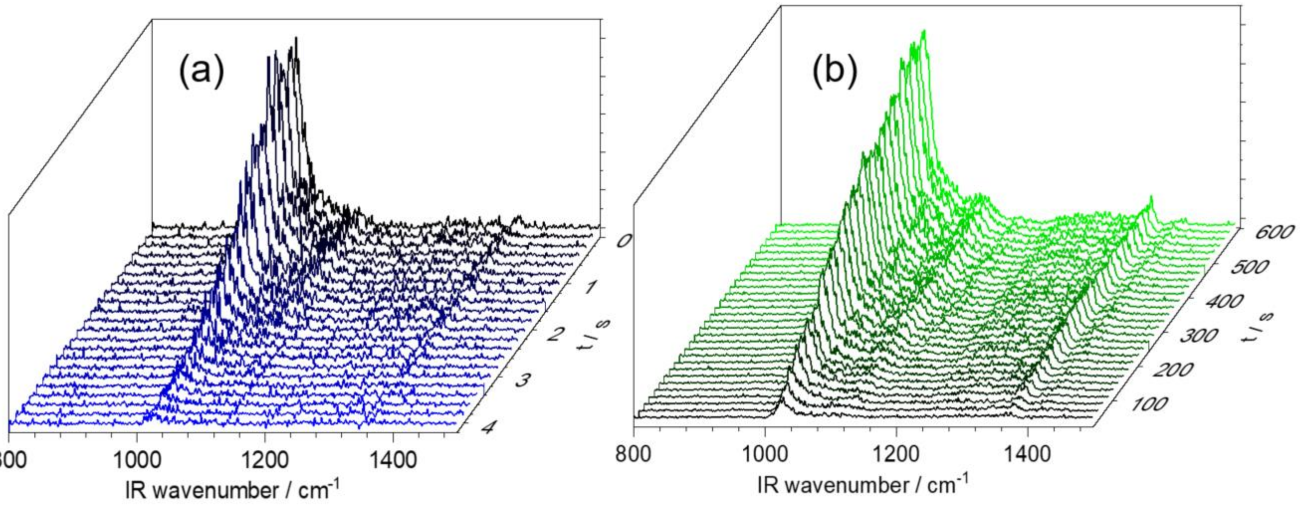

Figure S4: *SFG intensity of the symmetric S-O stretching vibration of butyl-AAP-C_4S at the air water interface as a function of time. (a) A 0.25 mM solution of the deuterated surfactant was equilibrated under 520 nm irradiation and at 0 s the irradiation was changed to 365 nm, leading to a fast decrease in signal intensity. (b) The same sample was first equilibrated under 365 nm irradiation, which was changed to 520 nm at t = 0 s.*

8. SFG Spectra of O-H and C-H stretching bands

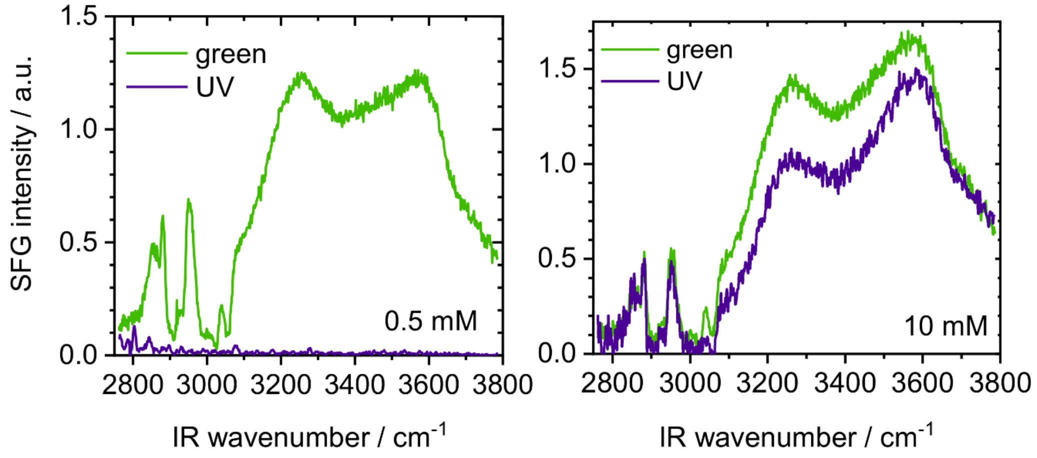

Figure S5: *SFG spectra of the air-water interface with 0.5 and 10 mM (as indicated in the figure) butyl-AAP-C_4S surfactant and at 520 nm green (green lines) and 360 nm UV irradiation.*

9. Fitting of Neutron Reflectometry Data

The data obtained from the measurements on FIGARO (10.5291/ILL-DATA.9-12-540) have been processed and fitted according to the work flow described by Campbell *et al.* [7] using Igor 8.02 and the Motofit Plug-In[8]. In short, this work flow involved four key points. First, the surfactant molecules were divided into two stratified layers parallel to the interface: one in contact with air comprising hydrocarbon chains, and one in contact with water containing solvated head groups. Second, a constraint was applied so that the number of chains in the upper layer matched the number of head groups in the lower layer in order to respect the true chemical structure of the surfactant. Third, the thickness of the hydrated head group layer was estimated from the physical molecular dimension of 4 Å. Fourth, equivalent inter-layer roughness was applied at all three interface (air/chains, chains/hydrated head groups and hydrated head groups/water) consistent with the presence of capillary waves. One exception to the workflow outlined in ref. 3 was that instead of a fixed volume fraction of 1 for the upper layer of chains, which has been shown to be appropriate for disordered surfactant monolayers of high coverage, air was allowed

into the layer and the volume fraction was varied due to the more rigid surfactant molecules with a double bond studied in the present work. As such, there were only two free fitting parameters: the volume fraction of the chains layer and the thickness of the chains layer. For the application of this fitting procedure, the surfactant molecules were split into a hydrophobic (red) and a hydrophilic part (blue), as shown in Scheme 1. The former consists of the AAP moiety together with the terminal butyl substituent as well as four methylene groups between the pyrazole and the sulfonate group, which makes up the hydrophilic part. Partial molar volumes for butyl, butylene and sodium sulfonate moieties were calculated from literature-known increments[9] while the molecular volume of the AAP core was determined from the density of a AAP derivative described elsewhere[10].

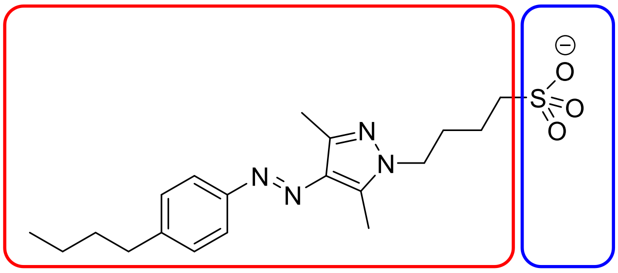

Scheme S1_Hydrophobic (red) and hydrophilic (blue) parts of the butyl-AAP-C$_4$S as used for the modelling approach._

Scattering length densities (*SLD*s)

$$SLD = \frac{\sum_{i=1}^{N} b_c}{V_m} \quad (1)$$

were calculated according to equation (1), where b_c are the bound coherent scattering length (SL)contributions, N is the number of atoms in a molecule and V_m is the molecular volume, are given in Table S1.

a)

Atom	b_c / fm
H	-3.7390
D	6.671
C	6.6460
N	9.36
O	5.803
S	2.847
Na	3.63

b)

	V_m / Å^3	*SL* / fm	*SLD* / 10^{-6} Å^{-2}
Hydrophilic part	45.98	23.886	5.19
Hydrophobic part (H)	503.4	63.331	1.26
Hydrophobic part (D)		198.661	3.95

Table S 1: _a) Bound coherent scattering lengths taken from ref.[11] b) calculated molar volumes and scattering length densities._

The parameters and values used for the aforementioned fitting of the data are represented in Table 2. Besides information about the samples the number of layers, SLDs for the different layers and the corresponding roughnesses are given as well as the thickness of the layers and the solvent penetration in the headgroup layer.

For the samples at the two higher concentrations, the thickness of the layer containing the hydrophobic chains was determined to be around 16 Å regardless of green or UV illumination. In contrast, the layer thickness in the case of 0.1 mM under UV illumination is extremely small and it should be noted that this result may arise from the formation of segregated patches of surfactant at the interface, which in turns can cause off specular scattering. Such an effect is consistent with the fact that the data lie above the model fit for this sample only. The volume fraction parameter increases under both illumination conditions with increasing concentration, representing a closer packing of the hydrophobic chains with less air incorporated. Please take into account that for the 0.1 mM sample under UV illumination it was not possible to distinguish between a model that incorporated air into the hydrophobic layer and one that does not as neither one showed significantly better results.

Conc. / mM	0.1 mM							
Illumination	Green / E conformation				UV / Z conformation			
Surfactant	d	h	d	h	d	h	d	h
Solvent	ACMW	ACMW	D_2O	D_2O	ACMW	ACMW	D_2O	D_2O
Subphase-SLD / 10^{-6} Å^{-2}	0	0	6.3	6,3	0	6.3	0	6.3
Background	$7{\cdot}10^{-7}$	$1.65{\cdot}10^{-6}$	$2{\cdot}10^{-7}$	$2{\cdot}10^{-7}$	$7{\cdot}10^{-7}$	$1.65{\cdot}10^{-6}$	$2{\cdot}10^{-7}$	$2{\cdot}10^{-7}$
Interlayer Roughness / Å	2.9				2.8			
Chain Thickness	16.195				2.7878			
eff. SLD Chains / 10^{-6} Å^{-2}	1.965	0.625	1.965	0.625	3.8907	1.2375	3.8907	1.2375
Volume Fraction Chains	0.5				0.99			
Headgroup Thickness / Å	4				4			
Headgroup SLD / 10^{-6} Å^{-2}	5.2				5.2			
Solvent Percentage in Headgroup Layer	82				95			

Conc. / mM	0.5 mM							
Illumination	Green / E conformation				UV / Z conformation			
Surfactant	d	h	d	h	d	h	d	h
Solvent	ACMW	ACMW	D_2O	D_2O	ACMW	ACMW	D_2O	D_2O
Subphase-SLD / 10^{-6} Å^{-2}	0	0	6.17	6.17	0	0	6.16	6.16
Background	$7{\cdot}10^{-7}$	$1.65{\cdot}10^{-6}$	$2{\cdot}10^{-7}$	$2{\cdot}10^{-7}$	$7{\cdot}10^{-7}$	$1.65{\cdot}10^{-6}$	$2{\cdot}10^{-7}$	$2{\cdot}10^{-7}$
Interlayer Roughness / Å	3.5				2.8			
Chain Thickness	16.557				15.671			
eff. SLD Chains / 10^{-6} Å^{-2}	2.6724	0.85	2.6724	0.85	1.179	0.375	1.179	0.375
Volume Fraction Chains	0.68				0.30			
Headgroup Thickness / Å	4				4			
Headgroup SLD / 10^{-6} Å^{-2}	5.2				5.2			
Solvent Percentage Headgroup Layer	74				89			

Conc. / mM	1.5 mM							
Illumination	Green / E conformation				UV / Z conformation			
Surfactant	d	h	d	h	d	h	d	h
Solvent	ACMW	ACMW	D_2O	D_2O	ACMW	ACMW	D_2O	D_2O
Subphase-SLD / 10^{-6} Å^{-2}	0	0	6.1989	6.1989	0	0	6.3	6.3
Background	$7\cdot10^{-7}$	$1.65\cdot10^{-6}$	$2\cdot10^{-7}$	$2\cdot10^{-7}$	$7\cdot10^{-7}$	$1.65\cdot10^{-6}$	$2\cdot10^{-7}$	$2\cdot10^{-7}$
Interlayer Roughness / Å	3.5				2.9			
Chain Thickness	17.67				16.681			
eff. SLD Chains / 10^{-6} Å^{-2}	2.5545	0.8125	2.5545	0.8125	1.7685	0.5625	1.7685	0.5625
Volume Fraction Chains	0.65				0.45			
Headgroup Thickness / Å	4				4			
Headgroup SLD / 10^{-6} Å^{-2}	5.3				5.3			
Solvent Percentage in Headgroup Layer	73				82			

Conc. / mM	6.0 mM							
Illumination	Green / E conformation				UV / Z conformation			
Surfactant	d	h	d	h	d	h	d	h
Solvent	ACMW	ACMW	D_2O	D_2O	ACMW	ACMW	D_2O	D_2O
Subphase-SLD / 10^{-6} Å^{-2}	0	0	6.3	6.3	0	0	6.15	6.15
Background	$7\cdot10^{-7}$	$1.65\cdot10^{-6}$	$2\cdot10^{-7}$	$2\cdot10^{-7}$	$7\cdot10^{-7}$	$1.65\cdot10^{-6}$	$2\cdot10^{-7}$	$2\cdot10^{-7}$
Interlayer Roughness / Å	3.6				4.4			
Chain Thickness	17.693				16			
eff. SLD Chains / 10^{-6} Å^{-2}	3.42	1.08	3.42	1.08	3.148	0.997	3.148	1.2375
Volume Fraction Chains	0.87				0.8			
Headgroup Thickness / Å	4				4			
Headgroup SLD / 10^{-6} Å^{-2}	4.52				4.155			
Solvent Percentage in Headgroup Layer	83				70			

***Table S2**: Overview on the parameters used for the fitting procedure of the neutron reflectivity data for both green and UV irradiation of four samples with concentrations of 0.1 mM, 0.5 mM, 1.5 mM and 6.0 mM. Fitted parameters are highlighted in blue. Sample information is stated as concentration, illumination conditions (GR or UV), isotopic labelling (h for hydrogenous and d for deuterated surfactant) as well as solvent (ACMW for air contrast matched water and D_2O: Key for labelling of the different layers: Chains: layer of the hydrophobic part of the molecules; Headgroup; the sulfonate anchor group of the surfactants Key for the different parameters: Background: Background values for the corresponding surfactant solvent combination; SLD: scattering length density; Thickness: layer thickness; Volume fraction: ratio of surfactant to air within the layer; interlayer roughness: surface roughness caused by capillary waves; solvent percentage: solvent penetration inside the layer. Summarizing the results from the fitting procedure mentioned above, Figure S6 shows the recorded data together with the obtained fits using the parameters listed in table 1 and 2.*

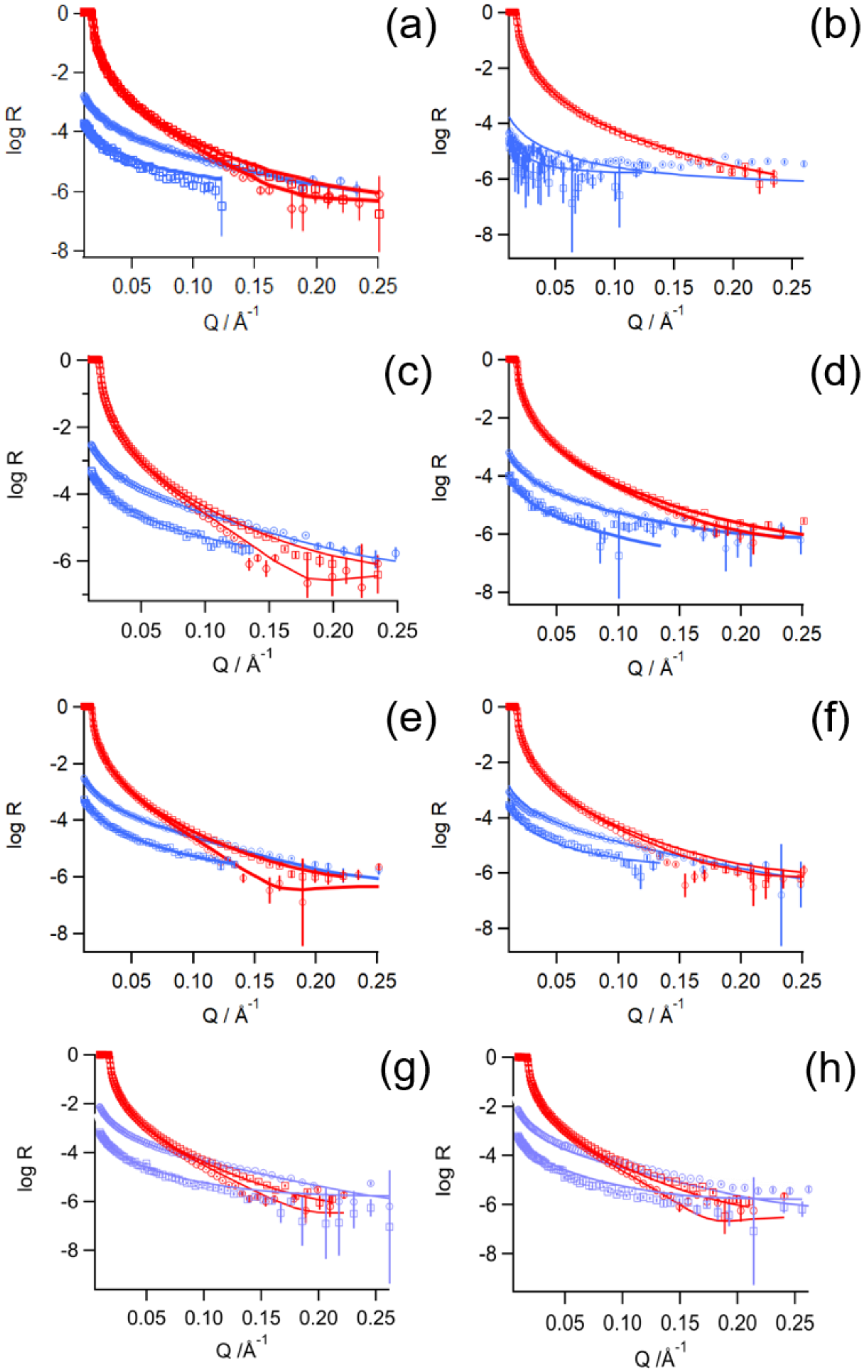

Figure S6: *Reflectivity curves for butyl-AAP-C$_4$S under both illuminations for 0.1 mM ((a) green, (b) UV), 0.5 mM ((c) green, (d) UV), 1.5 mM ((e) green, (f) UV) and 6.0 mM ((g) green, (h) UV). Squares represent non-deuterated surfactant, circles deuterated surfactant while symbols and lines in blue refer to ACMW and those in red to measurements in D$_2$O.*

10. Spectra of LEDs for sample irradiation

For further characterization of the LEDs used for illumination of the AAP-surfactants emission spectra were recorded (Figure S7).

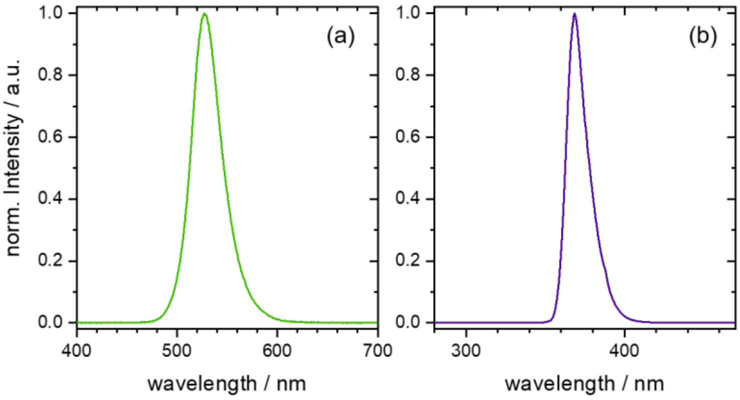

***Figure S7**: Normalized Emission spectra of both LEDs, green (a) and UV (b).*

11. References

[1] T. Kairaliyeva, E. V. Aksenenko, N. Mucic, A. V. Makievski, V. B. Fainerman, R. Miller, *J. Surfact. Deterg.* **2017**, *20*, 1225.

[2] *IsoFit Software: http://www.thomascat.info/Scientific/AdSo/AdSo.htm*.

[3] S. Hayashi, K. Hayamizu, *BCSJ* **1991**, *64*, 685.

[4] a) M. Piotto, V. Saudek, V. Sklenář, *J Biomol NMR* **1992**, *2*, 661; b) M. Liu, X.-a. Mao, C. Ye, H. Huang, J. K. Nicholson, J. C. Lindon, *J. Magn. Res.* **1998**, *132*, 125.

[5] C. E. Weston, R. D. Richardson, P. R. Haycock, A. J. P. White, M. J. Fuchter, *J. Am. Chem. Soc.* **2014**, *136*, 11878.

[6] N. García Rey, E. Weißenborn, F. Schulze-Zachau, G. Gochev, B. Braunschweig, *J. Phys. Chem. C* **2019**, *123*, 1279.

[7] R. A. Campbell, Y. Saaka, Y. Shao, Y. Gerelli, R. Cubitt, E. Nazaruk, D. Matyszewska, M. J. Lawrence, *J. Coll. Int. Sci.* **2018**, *531*, 98.

[8] A. Nelson, *J. Appl. Crystallogr.* **2006**, *39*, 273.

[9] a) J. T. Edward, *J. Edu. Chem.* **1970**, *47*, 261; b) A. Gavezzotti, *J. Am. Chem. Soc.* **1983**, *105*, 5220; c) Luciano Lepori, Paolo Gianni, *J. Solution Chem.* **2000**, *29*, 405.

[10] M. Schnurbus, L. Stricker, B. J. Ravoo, B. Braunschweig, *Langmuir* **2018**, *34*, 6028.

[11] V. F. Sears, *Neutron News* **1992**, *3*, 26.
